# Progression-Free but No Overall Survival Benefit for Adult Patients with Bevacizumab Therapy for the Treatment of Newly Diagnosed Glioblastoma: A Systematic Review and Meta-Analysis

**DOI:** 10.3390/cancers11111723

**Published:** 2019-11-04

**Authors:** Nagham Kaka, Karim Hafazalla, Haider Samawi, Andrew Simpkin, James Perry, Arjun Sahgal, Sunit Das

**Affiliations:** 1 School of Medicine, National University of Ireland Galway, H91 TK33 Galway, Ireland; N.Kaka1@nuigalway.ie; 2 Sidney Kimmel Medical College at Thomas Jefferson University, Philadelphia, PA 19107, USA; karim.hafazalla@jefferson.edu; 3 Li Ka Shing Knowledge Institute, St. Michael’s Hospital, Toronto, ON M5B 1A6, Canada; 4 Division of Hematology/Oncology, St. Michael’s Hospital, Toronto, ON M5B 1W8, Canada; SamawiH@smh.ca; 5 School of Mathematics, Statistics and Applied Mathematics, National University of Ireland, H91 TK33 Galway, Ireland; andrew.simpkin@insight-centre.org; 6 Department of Neurology, Sunnybrook Hospital, Toronto, ON M4N 3M5, Canada; james.perry@sunnybrook.ca; 7 Department of Radiation Oncology, Sunnybrook Hospital, Toronto, ON M4N 3M5, Canada; Arjun.Sahgal@sunnybrook.ca; 8 Division of Neurosurgery, University of Toronto, Toronto, ON M5B 1W8, Canada

**Keywords:** glioblastoma, bevacizumab, meta-analysis

## Abstract

Glioblastoma (GBM) is the most common high-grade primary brain tumor in adults. Standard multi-modality treatment of glioblastoma with surgery, temozolomide chemotherapy, and radiation results in transient tumor control but inevitably gives way to disease progression. The need for additional therapeutic avenues for patients with GBM led to interest in anti-angiogenic therapies, and in particular, bevacizumab. We sought to determine the efficacy of bevacizumab as a treatment for newly diagnosed GBM. We conducted a literature search using the PubMed database and Google Scholar to identify randomized controlled trials (RCTs) since 2014 investigating the safety and efficacy of bevacizumab in the treatment of adult patients (18 years and older) with newly diagnosed GBM. Only Level Ι data that reported progression-free survival (PFS) and overall survival (OS) were included for analysis. Random effects meta-analyses on studies with newly diagnosed glioblastoma were conducted in R to estimate the pooled hazard ratio (HR) for PFS and OS. Six RCTs met requirements for meta-analysis, revealing a pooled estimate of PFS HR suggesting a 33% decreased risk of disease progression (HR 0.67, 95% CI, 0.58–0.78; *p* < 0.001) with bevacizumab therapy, but no effect on OS (HR = 1, 95% CI, 0.85–1.18; *p* = 0.97). A pooled estimate of the mean difference in OS months of −0.13 predicts little difference in time of survival between treatment groups (95% CI, −1.87–1.61). The pooled estimate for the mean difference in PFS months was 2.70 (95% CI, 1.89–3.50; *p* < 0.001). Meta-analysis shows that bevacizumab therapy is associated with a longer PFS in adult patients with newly diagnosed glioblastoma, but had an inconsistent effect on OS in this patient population.

## 1. Introduction

Glioblastoma (GBM), a subgroup of diffuse gliomas, is the most common high-grade primary brain tumor in adults [1]. Standard therapy for GBM entails maximal safe surgical resection followed by radiotherapy (RT) with concurrent and adjuvant temozolomide (TMZ) [2,3]. Despite aggressive treatment, survival following diagnosis with GBM remains dismal: median survival is 14–16 months, with only a minority of patients surviving beyond two years [4,5]. There is a significant need to identify and assess other therapeutic options for these patients.

The synonymous finding of neoangiogenesis in GBM suggested a possible utility for anti-angiogenic therapies in this disease. The monoclonal anti-VEGF-A antibody, bevacizumab (BEV), was a particularly attractive candidate for study, as drug delivery was deemed necessary only to the abluminal surface of tumor-associated endothelial cells. While early studies examining BEV in patients with recurrent malignant glioma were promising [6,7,8], the results of multiple randomized control trials (RCTs) examining the role of BEV in the treatment of patients with newly diagnosed GBM failed to show a positive effect of BEV on overall survival (OS) [9,10,11]. As a result, the role of BEV in the treatment of patients with newly diagnosed and recurrent GBM remains unclear.

Newly diagnosed and recurrent forms of GBM differ from a molecular, genetic, and clinical standpoint. Transcriptional profiling allows the classification of GBM into classical, proneural, or mesenchymal subtypes [12]. The classical subtype is seen more commonly in newly diagnosed GBM, while the mesenchymal subtype appears more frequently in recurrent GBM. Furthermore, tumor recurrence is associated with the accumulation of new genetic mutations. TP53 mutations are more common in recurrent GBM, particularly in the proneural and mesenchymal subtypes [12]. Proneural subtypes also include, but are not limited to, isocitrate dehydrogenase-1 (IDH1)-mutant GBM. The vast differences between newly diagnosed and recurrent GBM necessitate the treatment of these diseases as separate entities.

To address the ambiguity of the role of BEV for the treatment of newly diagnosed GBM, we conducted a systematic review of RCTs of patients diagnosed with newly diagnosed GBM treated with BEV. In addition, we performed a meta-analysis to determine the effect of BEV on OS and progression-free-survival (PFS) in adult patients diagnosed with newly diagnosed GBM.

## 2. Results

### 2.1. Systematic Literature Review

**Figure 1:**
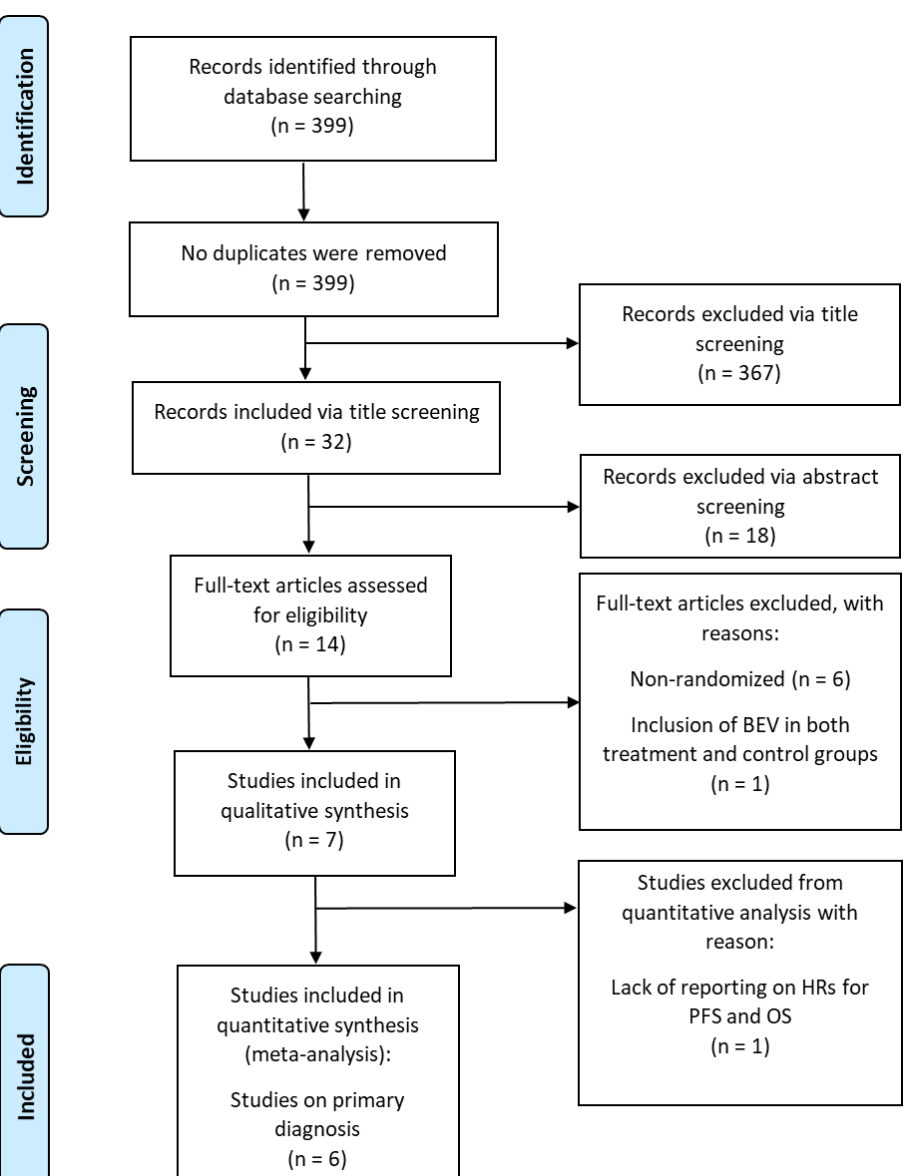
PRISMA flowchart of randomized controlled trials of patients with glioblastoma treated with BEV.

In total, 399 citations were retrieved from PubMed and Google Scholar. Screening for title led to the exclusion of 367 of these citations; the remaining 32 citations were screened for abstract. Of these, seven citations (n = 2,065 patients) met inclusion criteria and were eligible to be included in the qualitative synthesis of this literature review [9,10,13,14,15,16,17] (Table 1); the remaining 25 citations were excluded per the criteria detailed above. Each of the seven RCTs is specific to the primary diagnosis of GBM. Figure 1 shows the PRISMA flowchart. Treatment course varied widely in both the treatment and control groups across all seven RCTs. All studies administered BEV intravenously at 10 mg/kg every two weeks, starting at varying time points within treatment plan.

### 2.2. Data Extraction for Meta-Analysis

All seven RCTs investigating primary diagnosis of GBM included BEV solely in the treatment group. For OS HR, the study by Chauffert et al. was excluded due to inexplicable HR and CIs. For OS and PFS in months, the study by Chinot et al. was excluded from analysis due to a lack of CIs or standard errors (SEs) corresponding to the number of months. Finally, the study by Carlson et al. was excluded from all analysis due to missing HRs and corresponding CIs for both OS and PFS. This amounts to a total of six from the seven RCTs as eligible for a meta-analysis on the effect of the use of BEV for the treatment of newly diagnosed glioblastoma.

### 2.3. Meta-Analysis of Hazard Ratios

**Figure 2:**
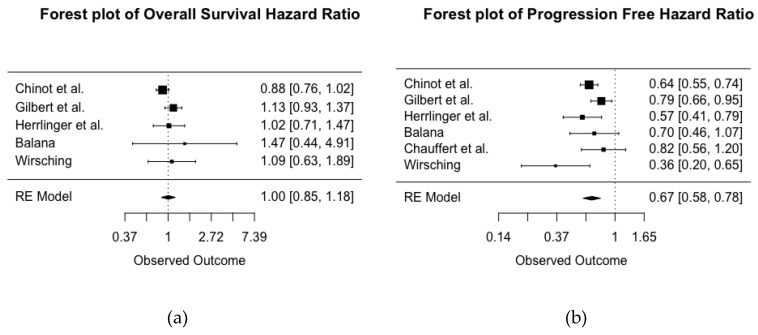
Forest plot of the pooled hazard ratios (HR) for overall survival (**a**) and progression free survival (**b**) across six randomized controlled trials (RCTs) of treatment for newly diagnosed glioblastoma with and without BEV. HR < 1 indicates a protective effect of BEV.

Figure 2a represents the pooled OS HR across five RCTs of primary diagnosis of GBM (total *n* = 1917) comparing treatment with and without BEV. The *I*^2^ statistic was 34%, suggesting a moderate between-study variability. A HR value below one indicates a protective effect of BEV. The pooled HR for OS was 1, which is not indicative of a protective effect of BEV on OS. The 95% confidence interval ranged from 0.85 to 1.18, with *p* = 0.97, which gives little to no evidence about a population effect of BEV on overall survival of patients with newly diagnosed GBM.

Figure 2b represents pooled PFS. Six RCTs of primary diagnosis of GBM reported on patients (total *n* = 2037) treated with and without BEV, with HR < 1 indicating a protective effect of BEV. Heterogeneity was lower for PFS, with an *I*^2^ statistic of 40.77%, suggesting a moderate level of between-study variability. The pooled estimate of the HR suggests that treatment with BEV is associated with a 33% decreased risk of disease progression (HR 0.67, 95% confidence interval 0.58–0.78; *p* < 0.001).

### 2.4. Meta-Analysis of Months

**Figure 3:**
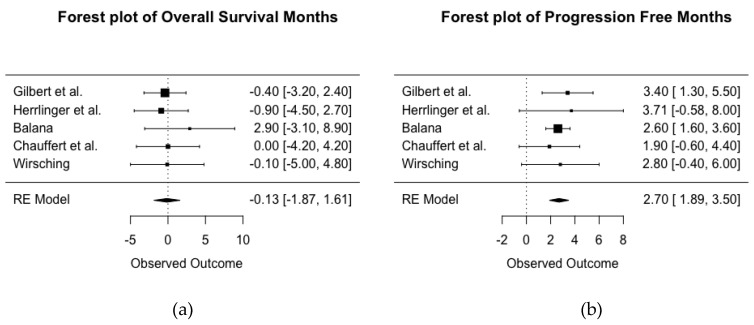
Forest plot of the pooled mean difference in months between treatment with and without BEV for overall survival (**a**) and progression free survival (**b**) across five RCTs of newly diagnosed glioblastoma. HR < 1 indicates a protective effect of BEV.

Five RCTs’ (total *n* = 1116) reported data that allowed for analysis of mean difference OS between treatment with and without BEV (Figure 3a). There were five RCTs with primary diagnosis of GBM available for comparison, with a larger number of the mean difference in months indicating a protective effect of BEV. The *I*^2^ statistic of 0% suggests the studies are homogenous. A pooled estimate of the mean difference in months of overall survival of −0.13 suggests very little difference between treatment groups in duration of survival post-diagnosis with GBM (95% CI for mean difference in months is −1.87 to 1.61).

Figure 3b shows a forest plot of the mean difference in months of PFS between treatment with and without BEV across five RCTs of primary diagnosis of GBM (total *n* = 1116). An *I*^2^ statistic of 0% suggests the studies are homogenous. The pooled estimate for mean difference in the months of PFS was 2.7 (95% CI, 1.89–3.50; *p* < 0.001), suggesting patients with newly diagnosed GBM treated with BEV had an added mean of 2.7 months of PFS in comparison to those not receiving BEV.

## 3. Discussion

The use of BEV for the treatment of GBM has been shown to prolong PFS; its effect on OS, however, has been less clear [11,18]. Meta-analysis has previously proven valuable as a knowledge translation guide in the setting of inconclusive primary evidence (see, for example, effect of pooled analysis of data from the DECIMAL, DESTINY and HAMLET studies on the management of patients with malignant infarction of the middle cerebral artery) [19]. Our aim was to review the recent literature on the use of BEV for the treatment of newly diagnosed GBM. The data allowed us to run meta-analyses on the HR and months of OS and PFS as a measure of survival.

The literature search yielded seven RCTs investigating the efficacy of BEV on the treatment of newly diagnosed GBM, six of which had published sufficient data to run the meta-analyses. Our meta-analysis showed that BEV did not have a significant effect on OS; however, it conferred a significantly prolonged PFS. The secondary meta-analysis on the mean difference in the number of months between the treatment and control group provided confirmatory results to our original meta-analysis on HRs. These findings are consistent with the previous literature stating that BEV does not provide a protective effect in terms of OS in patients with newly diagnosed GBM, despite its prolongation of PFS [11].

The use of OS and PFS as primary endpoint measures in evaluating a therapeutic treatment with respect to tumor progression has long been considered the gold standard of oncology studies. Although PFS is not a direct measure of survival of patients diagnosed with the disease, when compared to OS, it classically has a similar magnitude and direction for improvement of disease progression [20]. The findings of this study challenge this well-established relationship between OS and PFS. One possible explanation for the failure of BEV to improve OS despite prolongation of PFS is that BEV therapy might result in changes in imaging findings that do not necessarily correlate with beneficial effects on tumor biology. It is known that BEV therapy results in a decrease in contrast enhancement on MRI [21]; whether this imaging finding correlates with tumor response is less clear. In fact, some authors have speculated the BEV could impair the beneficial effects of pseudoprogression, which has been thought to depend on blood–brain barrier permeability [22]. Conversely, multiple studies in patients with recurrent GBM have found radiographic response to correlate with OS [23,24,25].

One of the seven RCTs was not included in the meta-analysis due to a lack of confidence intervals, but their findings are of appreciable value to this systematic review. This study, by Carlson et al., compared the treatment of newly diagnosed GBM with hypo-IMRT plus TMZ with or without BEV. Findings showed no improvement in OS, and little but statistically insignificant improvement in PFS [14].

BEV could have a role in the treatment of particular subgroups of patients with newly diagnosed GBM. Several recent trials have investigated the effects of BEV as a treatment option for patients harboring either methylated or non-methylated O^6^-methylguanine-DNA methyltransferase (MGMT) promoters. MGMT promoter status has long been proposed as an indicator for prognosis and treatment planning. The phase III Avastin in Glioblastoma (AVAglio) and RTOG 0825 trials provided much insight into this question [9,10]. Both studies assessed the survival benefits of RT and TMZ in conjunction with either BEV or a placebo in newly diagnosed GBM. The RTOG 0825 group found overall MGMT status was a prognostic indicator in all treatment groups [10]. MGMT unmethylated patient overall survival was 14.3 months (95% CI, 13.6–15.3) versus 23.2 months (95% CI, 20.1–28.3) for methylated status patients (HR, 2.10; 95% CI, 1.65–2.68; *p* < 0.001). Median PFS was 8.2 months (95% CI, 7.5–9.2) for MGMT unmethylated tumors versus 14.1 months (95% CI, 10.5–16.1) for methylated tumors (HR, 1.67; 95% CI, 1.36–2.05; *p* < 0.001). Of note, BEV was not found to improve survival in this patient subset. Similarly, AVAglio found significant prolongation using BEV with respect to PFS for both methylated and unmethylated MGMT status, but no significant difference in OS between the two subgroups [9].

More recently, the GLARIUS trial assessed BEV with irinotecan versus temozolomide in newly diagnosed nonmethylated MGMT GBM [16]. The GLARIUS trial found an increase in PFS with BEV therapy, increasing from a median of 5.99 months (95% CI, 2.7–7.3 months) with TMZ to 9.7 months (95% CI, 8.7–10.8 months; *p* < 0.001) with BEV and irinotecan. As with prior trials, they also reported a nonsignificant OS result: 16.6 months (95% CI, 15.4–18.4 months) with BEV+IRI compared to 17.5 months (95% CI, 15.1–20.5 months) with TMZ. They did note, however, that, with rank-preserving structural failure time analysis, a significant OS benefit with BEV was found. This finding suggested that BEV crossover could have confounded OS results for the TMZ group. The results of these trials correspond with our findings that BEV therapy for patients with newly diagnosed GBM prolongs PFS, particularly in those with MGMT methylation, but OS remains unchanged.

Glioblastoma molecular subtype has also been a point of much discussion. Specifically, the mesenchymal and proneural glioblastomas have been used as proposed prognostic markers. The mesenchymal variants have higher VEGF/angiogenic marker expression when compared to their proneural counterparts [26]. With VEGF posing as a possible therapeutic target, Sandmann and colleagues retrospectively assessed the AVAglio database in order to evaluate the effect of BEV on PFS and OS depending on molecular subtype. They classified patient subtype based on the criterion by Phillips et al. [27]. After excluding *IDH1* mutation-positive samples, they found proneural tumors had a median OS of 12.2 months versus 17.4 months in the mesenchymal subtype when treated with placebo (*p* = 0.408). On univariate analysis in patients treated with BEV, PFS was significantly increased in proneural (9.9 vs. 5.7 months; HR 0.57; 95% CI, 0.37–0.89; *p* = 0.036) and mesenchymal (10.1 vs. 5.8 months; HR 0.57; 95% CI, 0.40–0.82; *p* = 0.0076) tumors. Counterintuitively, they found BEV provided a significantly improved OS benefit when compared to placebo in proneural tumors (17.1 vs. 12.8 months; HR 0.63; 95% CI, 0.41–0.99; *p* = 0.045), but not mesenchymal tumors (17.2 vs. 17.4 months; HR 0.98; 95% CI, 0.67–1.45; log-rank *p* = 0.929). This was also the case in multivariate analysis, with BEV in addition to radiotherapy plus TMZ conferring significant OS advantage for *IDH1* wild-type proneural tumors (HR 0.42; 95% CI, 0.25–0.71; *p* = 0.001), but not non-proneural *IDH1* wild-type tumors (HR 1.00; 95% CI, 0.74–1.36; *p* = 0.985). This challenges the current dogma of anti-angiogensis therapy, whereby mesenchymal tumors hypothetically should have benefited more due to BEV treatment. However, Lambrechts and colleagues argued that the expression of VEGF plasma markers did not correlate consistently with BEV efficacy in a variety of cancers [28]. Additionally, Sandmann and colleagues argued the poorly differentiated mesenchymal tumors could allow the tumor to develop a resistance to BEV over time, whereby there would be an initial response to therapy that leads to the improvement in PFS before resistance is conferred, leaving OS unchanged [26]. Overall, this study indicated BEV is a promising agent when used with the standard TMZ and RT for a survival benefit in proneural tumors.

The advent of next generation sequencing has additionally provided opportunities to further characterize the effects of BEV on the tumor environment in glioblastoma. A study by Adilijiang and colleagues assessed the impact of BEV alone and in conjunction with temozolomide in the treatment of both newly diagnosed *IDH1* wildtype and mutant glioblastoma [29]. Using RNA-seq, they found that treatment with BEV and TMZ results in the upregulation of certain microenvironment related genes in *IDH1* mutant tumors in vitro, specifically those involving immune response and extracellular matrix organization. Additionally, they found that expression of genes involved in cell-cycle progression was reduced. These findings were accompanied by suppression of tumor growth in xenograft models. This benefit was not seen in the *IDH1* wildtype variants both in vitro and in vivo. These findings are promising and provide evidence for the use of BEV in *IDH1* mutant glioblastomas.

The effect of BEV on baseline quality of life was discussed in three studies included in the meta-analysis [9,10,16]. Quality of life was self-reported using a validated core quality-of-life questionnaire (EORTC QLQ-C30) and a second questionnaire specifically for patients with brain tumors (QLQ-BN20). Chinot and colleagues found that, despite the rate of adverse events being higher in the BEV group, the quality of life was maintained at baseline for longer in the BEV group [9]. Conversely, Gilbert and colleagues found the BEV group had greater deterioration in perceived severity of symptoms, an increased symptom burden, and a worse quality of life [10]. Herrlinger and colleagues found no significant difference between patients in the BEV group and those in the placebo group with respect to quality of life [16]. As such, the literature is indeterminate on the impact of BEV on quality of life in patients diagnosed with newly diagnosed glioblastoma.

Our study has multiple limitations including the limited availability of RCTs on GBM in the literature, along with the lack of reporting on data deemed necessary to run a meta-analysis on the findings; therefore, we were limited to only six RCTs undergoing the meta-analysis. Further, there were differences in treatments received amongst the patients enrolled in the seven RCTs identified, which introduces variation across studies in which the effects of all these combined treatments cannot be dissociated from that of BEV, ultimately rendering it difficult to find a common ground with respect to treatment protocols across all the RCTs. Lastly, the number of participants per study differed widely, ranging from 56 to 921. This means the sample sizes were heterogenous across trials, introducing further stochasticity to our results.

While BEV has been studied extensively for newly diagnosed glioblastoma, there is still much to be understood regarding the antibody’s effect on the blood–brain barrier and tumor microenvironment. A recent study assessing perfusion and permeability in concomitant TMZ/BEV delivery for recurrent GBM showed that vascular permeability decreased after BEV administration, with a corresponding compromise in TMZ delivery [30]. These findings provide a possible explanation to the lack of OS improvement seen in many BEV trials. The authors suggested perhaps investigating a lower dose of BEV could be of benefit, so as to better balance the effects of reduced tumor angiogenesis while maximizing TMZ delivery. Additionally, it would be interesting to determine if BEV has a role as an adjunct to novel therapies, such as magnetic tumor-treating fields and laser interstitial therapy [31].

## 4. Materials and Methods

### 4.1. Systematic Literature Review

This meta-analysis was conducted according to PRISMA guidelines. A literature search using PubMed and Google Scholar was conducted by NK and KH. Search terms for PubMed entailed “primary glioblastoma” AND “BEV”. Search terms for Google Scholar included “primary glioblastoma” AND “BEV” with exclusion filters for the terms “recurrence”, “recurrent”, “progression”, and “progressive”. All studies meeting search criteria were then independently reviewed and checked by KH. Date range for selected articles was set between “01 Monday 2014” to “31 December 2018”, and the option “human studies” was chosen to narrow down the search results. The title of the citations was screened first, followed by the abstract, and lastly the full article. Only Level I articles, categorized as properly powered and conducted randomized-control trials, were selected. Inclusion criteria for the systematic review and meta-analysis were as follows: written in English, consisted of adult patients (18 years of age and older) with newly diagnosed glioblastoma, progression-free survival (PFS) and overall survival (OS) as primary or secondary end points, and a treatment course that included BEV.

The meta-analysis included studies that had BEV in the treatment group only, whereas the qualitative analysis included studies with any treatment course containing BEV; it includes studies in the treatment group only and studies with BEV in both the treatment and control groups. Exclusion criteria for the systematic review and meta-analysis constituted non-English written studies, retrospective study design, lack of reporting on OS and PFS, studies on pediatric patients, non-RCT studies, and unclear differentiation of survival data based on treatment or tumor-subtype. Additional exclusion criteria for the meta-analysis consisted of the use of BEV in both the treatment and control groups, studies investigating recurrent GBM, and lack of sufficient data reporting on OS and PFS to run the meta-analysis.

### 4.2. Data Extraction

Variables extracted from papers included study design, patient population demographics, histology and molecular subtype, adverse effects, and PFS and OS months with their corresponding hazard ratios. The 95% confidence interval (CI) of the PFS and OS hazard ratios was reported for use as input in the meta-analysis. All data were extracted into another predefined Excel workbook. Survival outcomes included OS and PFS months and hazard ratios, with their corresponding 95% CIs. Article selection eligibility was cross-referenced and verified by coauthors.

### 4.3. Statistical Analysis

Random effects meta-analyses were conducted to estimate the pooled hazard ratio of OS and PFS across studies of newly diagnosed GBM that used BEV to the treatment group only. Restricted maximum likelihood was used to estimate the heterogeneity variance. Hazard ratios (HRs) and their 95% CIs were obtained from each study, and the standard errors (SEs) were calculated from the 95% CI limits provided in each paper [32,33,34]. First, the HRs and their corresponding CIs were back transformed to the log scale. The SEs were calculated using the following formula:

log HR ± 1.96 × SE = log UL/LL. UL and LL refer to the upper and lower limits of the 95% CI, respectively. For our example, this would be 0.372 − 1.96 × SE = −0.128 and 0.372 + 1.96 × SE = 0.871. The SE can then be calculated from this formula, e.g. (0.872 − 0.372)/1.96 = 0.255.

A secondary analysis focused on the median OS and PFS months for RCTs reporting on primary diagnosis of GBM. The same formula above was used to calculate the SE from the reported 95% CIs of the OS and PFS months:

log M ± 1.96 × SE = log UL/LL. “M” here refers to the mean difference in the number of months of OS and PFS between groups with and without use of BEV.

Heterogeneity between studies was measured using the *I*^2^ statistic [33], where a higher *I*^2^ indicates increased between-study heterogeneity, and using low, moderate, and high to *I*^2^ values of 25%, 50%, and 75%, respectively [35]. Meta-analysis was performed using the metafor package in R v3.5 (R Foundation for Statistical Computing, Vienna, Austria. URL https://www.R-project.org/) to combine findings across the studies of primary diagnosis of glioblastoma reporting on PFS and OS hazard ratios, months and their corresponding standard errors [36,37].

## 5. Conclusions

Despite the limitations of this study, these findings enable evidence-based decision making regarding the use of BEV for the treatment of newly diagnosed GBM. Our study confirms the previous literature concluding that BEV therapy is associated with a prolonged PFS in adult patients diagnosed with newly diagnosed GBM, but has an inconsistent effect on OS. Future research is necessary to define a patient population for whom BEV therapy at diagnosis is indicated.

## Figures and Tables

**Table 1:** Randomized control trials since 2014 assessing bevacizumab use in newly diagnosed glioblastoma.

	First Author (Year)	Level of Study	*n*	Median Age (Years)	Duration of Follow-up (Months)	Treatment	Control	Prior Treatment
**1**	Balana [14] (2016)	I	Tx: 49 Control: 53	Tx: 62.9 Control: 62.0	>18.0	BEV + TMZ	TMZ	Biopsy only
**2**	Carlson [15] (2015)	I	Tx: 30 Control: 26	Tx: 56.5 Control: 60.5	Tx: 14.7 Control: 13.9	BEV + Hypo-IMRT + TMZ	Hypo-IMRT + TMZ	Biopsy/Sx
**3**	Chauffert [16] (2014)	I	Tx: 60 Control: 60	Tx: 60.2 Control: 60.9	–	BEV + IRI + RT + TMZ	RT + TMZ	Biopsy only
**4**	Chinot [9] (2014)	I	Tx: 458 Control: 463	Tx: 57.0 Control: 56.0	Tx: 14.4 Control: 13.7	BEV + RT + TMZ	PLA + RT + TMZ	Biopsy/Sx
**5**	Gilbert [10] (2014)	I	Tx: 312 Control: 309	Tx: 59.0 Control: 57.0	20.5	BEV + RT + TMZ	PLA + RT + TMZ	Biopsy/Sx
**6**	Herrlinger [17] (2016)	I	Tx: 116 Control: 54	Tx and Control: 56.0	–	BEV + IRI + RT	RT + TMZ	Biopsy/Sx
**7**	Wirsching [18] (2018)	I	Tx: 50 Control: 25	Tx: 70 Control: 70	–	BEV + Hypo-RT	Hypo-RT	Sx, steroids

Abbreviations: Tx, treatment; BEV, bevacizumab; TMZ, temozolomide; RT, radiotherapy; Sx, resective surgery; Hypo-IMRT, hypofractionated-intensity modulated radiotherapy; IRI, irinotecan; PLA, placebo; hypo-RT, hypofractionated radiotherapy. All studies administered BEV intravenously at 10 mg/kg every two weeks, starting at varying time points within treatment plan.
